# Dental Infections: Their Diagnosis and Treatment
*Paper read at a meeting of the Bath and Bristol Branch of the British Medical Association, 29th January, 1930.


**Published:** 1930

**Authors:** G. F. Fawn

**Affiliations:** Lecturer in Dental Surgery, University of Bristol. Assistant Dental Surgeon, Royal Infirmary, Bristol


### DENTAL INFECTIONS: THEIR DIAGNOSIS AND TREATMENT.*

BY

G. F. Fawn, M.R.C.S., L.R.C.P., B.D.S., L.D.S.,

*Lecturer in Dental Surgery, University of Bristol.*
*Assistant Dental Surgeon, Royal Infirmary, Bristol.*

---

At one time a paper with such a title would hardly be expected to appear on the programme of a Medical Society, but in these days, when the close relationship between dental infection and systemic disease is so well established, I thought it might be of interest to give some account of the conditions found by the dental surgeon which may be responsible for disease generally or locally. Really it is no more unreasonable to consider dental conditions at a medical meeting than it is to consider, say, eye conditions.

Dental infection may cause systemic disease by direct extension into adjacent structures, or by metastasis to distant parts of the body. Infection may spread from a tooth into the alveolar process, producing a diffuse osteomyelitis; into the soft tissues, producing a cellulitis; or into a neighbouring lymph gland, producing a lymphadenitis.

The maxillary sinus is not infrequently infected from the upper molars or pre-molars. Occasionally a

* Paper read at a meeting of the Bath and Bristol Branch of the British Medical Association, 29th January, 1930.

sinus thrombosis may result from the direct extension upwards of an acute dental infection, and very rarely a true septicæmia may result.

It is true that dental infection occurs with great frequency in comparison with the incidence of proved systemic disease of focal origin.

Many individuals, doubtless, carry infected teeth throughout life without showing manifest disease of focal origin, although marked injury of vital organs may result from the long-continued absorption of poisons without being recognized. Myocardial disease, which is responsible for such a high proportion of deaths after middle age, is an excellent example. The relation of focal infection to such lesions is often lost sight of. On the other hand, not infrequently disease due to other conditions has been incorrectly attributed to focal infection.

Now let us consider what are the sources of dental infection. They may be divided into four groups: pyorrhœa, devitalized teeth, retained roots of teeth, and residual sepsis.

As to *pyorrhœa*, it must be emphasized that the condition locally consists of an inflammatory condition which slowly extends into the spongy alveolus and, therefore, in time into the jaw proper, giving rise to residual sepsis. At the same time the pulps of the teeth become infected and a blood infection is added to an infection by surface extension. As Sir Frank Colyer remarks, it is not safe to judge the extent of this disease from clinical appearances only, and it is necessary to call in the aid of X-rays in order to ascertain how far bone destruction has proceeded. The gums may appear healthy, yet there may be an extensive disease of the alveolar process around the roots of the teeth and considerable bone destruction, the involved area being heavily infected with pathogenic streptococci.

The grave clinical effects resulting from infected bone are well known, and it is highly probable that the general clinical effects produced by dental infections are accounted for by the extent and nature of the disease of the bone in the neighbourhood of the teeth, rather than by disease in the gums or the teeth themselves. "It cannot be insisted upon too strongly that in every case of illness in which the teeth may be primarily responsible, even if the external appearances of the teeth and gums are healthy, a radiographic examination should be made to ensure that the alveolar process around the teeth is also healthy." The important point to remember is that even in the early stages of the disease there is an extension of infection to the stomach and bowel. Normally organisms swallowed are dealt with by the gastric juice, but in certain conditions of achlorhydria the duodenum first and then the lower intestine become infected from the mouth. The importance of this point is, that as in the past many people have lost their teeth without deriving any improvement of the condition from which they are suffering, it may possibly be explained on this theory, that the infection has extended to the colon, and removal of the teeth can have but little effect. Thus failures in obtaining a cure or improvement by removing the infective focus should only emphasize the importance of the early removal of dental infection. We should prevent the development of systemic disease rather than attempt to cure it after it has developed.

The next division of oral sepsis to be considered is that caused by *devitalized teeth*, or those teeth in which the dental caries has extended so deeply that the cavity is either too sensitive to stand filling or so deep that the pulp has already become infected. In these cases the pulp is anæsthetized and extracted, and an attempt is then made to clean and sterilize the canal and to fill it. The endings of the canals are so tortuous and narrow that it is impossible to fill these canals aseptically with any certainty. Infection is shut in and is pumped into the bone and the blood-stream.

Opinions are divided as to which of these two conditions, pyorrhœa or devitalized teeth, is more responsible for systemic disease.

The next division is that of *retained roots or fragments of teeth*, or *impacted unerupted teeth.* It is estimated that in cases of completely edentulous jaws, 25 per cent. are found on careful X-ray examination to present one or more tooth remnants.

Fourthly, there is *residual sepsis.* This condition has recently come into prominence since the frequent use of dental X-ray examination. It is only reasonable that such areas of residual sepsis should be found when one considers the technique of tooth removal.

It may be well now to describe what constitutes a thorough dental examination, and for the time being I am considering only those patients who have to all intents and purposes taken care of their teeth, not those whose mouths are in a terrible state from neglect. It is in these patients that we shall more commonly find peri-apical infection, but in these cases also it is extremely difficult for the dental surgeon to advise extraction unless there is some chance of the patient benefiting. The first stage of the examination is to test all teeth for vitality. This is done by subjecting each tooth to hot and cold stimuli. Even non-carious teeth may have dead pulps. Then all areas of the jaw must be X-rayed, whether edentulous or not. It will then be seen which teeth have been devitalized by the dental surgeon and root-filled, while those which have become dead through infection or injury, and any fragments of teeth, or areas of residual sepsis, will also be disclosed. It is impossible for anyone, even a dental surgeon, to give a satisfactory report on the mouth by mere inspection, so all patients should have an X-ray examination. Time is too short for me to discuss the question of devitalized teeth which show changes in the X-ray, and those which apparently show no changes. It will be sufficient to say that those which show no alteration in the bone have usually been found to be more harmful than those which show changes, the explanation being that abnormalities seen in the bone are frequently the result of resistance put up by the patient.

The prospect of benefit by dental treatment is so uncertain that it is only fair to the patient that no improvement in the general condition be promised, and yet it is reasonable to suppose that removal of dental sepsis must lessen the load of the individual patient. The earlier the dental sepsis is discovered the more hope of benefit. Blood examinations in diagnosis are of very little assistance, and it is unfortunate that at present there is little or no means of establishing a dental responsibility. Leucopenia and leucocytosis may either of them be found. The former condition is to be regarded as a danger signal, and in such cases extractions should always be few and comparatively far between. However, to my mind it is always advisable to go slowly in the matter of extractions. Any reaction following extraction is stated to be hopeful of benefit. This is particularly true of eye conditions. In all cases improvement in the general condition should not be expected too quickly. In arthritic conditions particularly improvement is long delayed.

Nothing that has been stated in the discussion of the treatment of infected teeth is to be interpreted as over-emphasizing dental infection in comparison with other foci of infection, and in studying patients with systemic disease of focal origin, all possible foci of infection should have careful clinical consideration. It is often apparent that serious damage results from focal infection without being recognized early enough to obtain satisfactory results from removal of the focus.

These facts justify the serious consideration of dental infection as a factor in the causation of disease, with special reference to its complete and early removal.

